# Experimental dataset of the impact assessment of vegetable intercropping on agroeconomic performances, pests and beneficials, and soil resources

**DOI:** 10.1016/j.dib.2023.109607

**Published:** 2023-09-24

**Authors:** B. Perrin, C. Leroy, L. Parès, P. Pradere, M. Goude, B. Salvador, T. Marrec, L. Comes, R. Huot-Marchand, E. Guillot, A. Lefèvre

**Affiliations:** Agroecological Vegetable Systems Experimental Facility, INRAE, Mas Blanc, 66200, Alénya, France

**Keywords:** Row intercropping, Greenhouse, Sweet pepper, Economic analysis, Soil resources, Arthropoda, Arbuscular mycorrhizal fungi, Organic farming

## Abstract

The data presented in this article were collected in the field at an experimental station in southern France under a Mediterranean climate. Experiments were conducted under three plastic walk-in tunnels used as blocks with organic farming practices over two successive years in a completely randomized design. The aim was to compare the intercropping of sweet pepper with basil, onion, lettuce, parsley or French bean to a sole crop of sweet pepper used as a control. The dataset provides information on cultural practices with details on inputs and working times used to estimate economic costs. The data also describe the climatic conditions under tunnels as well as the dynamics of soil nitrate concentration and water tension over time through treatments. Yields, economic benefits and the rates of products with visual defects are presented. In addition, some variables applied exclusively to sweet pepper crops, namely nitrate concentration in petiole sap, growth parameters, abundance of aerial pests and beneficials, incidence of root necrosis, arbuscular mycorrhizal fungi colonization rates and diversity in roots. The field dataset is made publicly available to allow free and easy access for the scientific and professional community to enable analysis and reuse. This is an open access article under the CC BY license (http://creativecommons.org/licenses/by/4.0/).

## Graphical abstract

**Figure:**
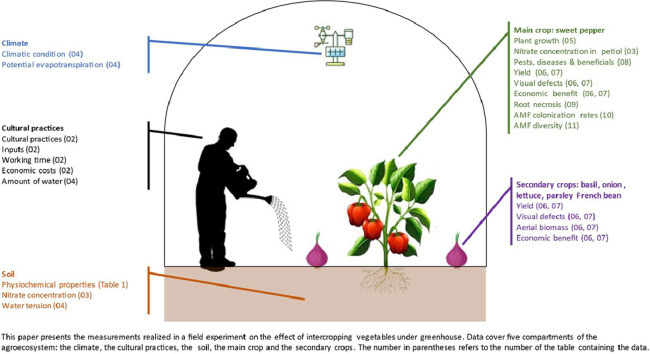

Specifications Table

**Table:** 

Subject	Biological and agricultural sciences, Agronomy and Crop Science, Soil Science
Specific subject area	Experimental dataset of sweet pepper intercropped under greenhouse: cultural practices, economic benefits, soil resources, yield, pests and beneficials
Type of data	Table Image Figure
How the data were acquired	Data were collected directly from experimental fields that underwent six treatments. The data included crop management information, field measurements and laboratory measurements. Please see the file “Variables description.csv” for more details on instruments, methods and protocols used for collecting data.
Data format	Raw Synthetized
Description of data collection	The data were collected in the field at an experimental station in southern France under a Mediterranean climate. The experiment was implemented with organic farming practices over two successive years (2021, 2022) in a completely randomized block design with three replicates, each one deployed in a 400 m² tunnel. Six treatments were randomly assigned to plots each year: sweet pepper intercropped with basil, onion, lettuce, parsley, French bean and a sole crop of sweet pepper (control).
Data source location	• Institution: INRAE • City/Town/Region: Alénya, Occitanie • Country: France • Latitude and longitude (42.637465, 2.971837) for collected samples/data: (42°38′14.9″N; 2°58′18.7″E; alt. 8.5 m)
Data accessibility	Repository name: https://recherche.data.gouv.fr Data identification number: 10.57745/QXZRDX Direct URL to data: https://entrepot.recherche.data.gouv.fr/dataset.xhtml?persistentId=doi:10.57745/QXZRDX

## 1 Value of the Data

• The data presented describe an original broad range of parameters (agronomical, ecological and economical) collected simultaneously from an agronomic experiment on intercropping in protected vegetable production systems. A sole crop of sweet pepper is compared with five promising intercropping systems on the same site. The secondary crops tested belong to five different botanical family and have therefore potentially varied effects on response variables. Datasets on these types of systems are scarce. They generally test only a single intercropping system and focus on one specific performance, organism or process.
• The experimental design is robust, with three replicates arranged in blocks, repeated two successive years. The methodology is relatively simple, fast and easily replicable. A detailed description of the pedoclimatic conditions of the trial provides information on the validity domain of the data.
• These data can be used by researchers working on intercropping systems, their design, their performances and the ecological processes at work. It is also a fully documented support resource for advisors, farmers, teachers or students looking for technical and economical references on intercropping systems.
• These data are helpful for designing high-performance vegetable intercropping systems or experiments on intercropping. They allow users to identify suitable crop combinations that perform better based on their yields, working times or economic returns and to address performances trade-offs. They are useful for quantifying and comparing services provided by different secondary crops to sweet pepper in terms of pest and disease regulation, reduction of damages on fruit and stimulation of beneficials. They can be used to reveal how different secondary crops compete with sweet pepper for nitrate and water, in a dynamic way and to identify weather and when and they limit crop production. They can also be mobilized as a basis for agroecological vegetable production system modeling, for metanalysis or as references to compare results from other studies.

## 2 Objective

This dataset was generated to fill a knowledge gap on intercropping vegetables under greenhouse in the context of French organic farms. The experiments were designed to reveal the agroeconomic advantages and drawbacks of introducing different secondary cash crops into a main crop and to clarify biological explanations.

## 3 Data Description

The dataset [1] encompass several measurements presented in 11 csv sheets and one table; additional figures were provided to facilitate data comprehension (Fig. 1, Fig. 2, Fig. 3, Fig. 4, Fig. 5, Fig. 6) and to describe the experimental design (Figs. 7–10).

**Fig. 1:**
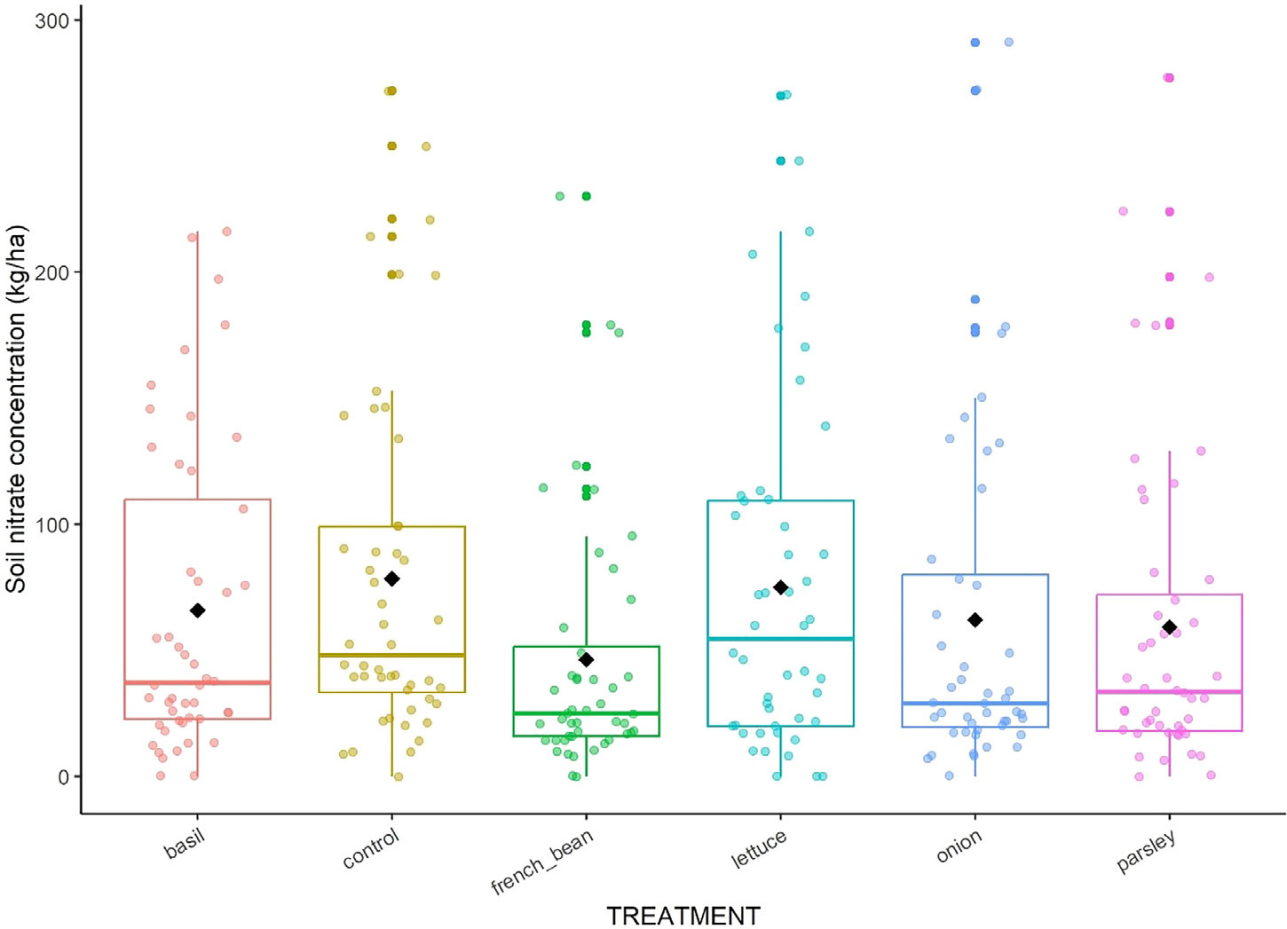
Boxplot of soil nitrate concentration in the six treatments. Data were pooled over time (days and years).

**Fig. 2:**
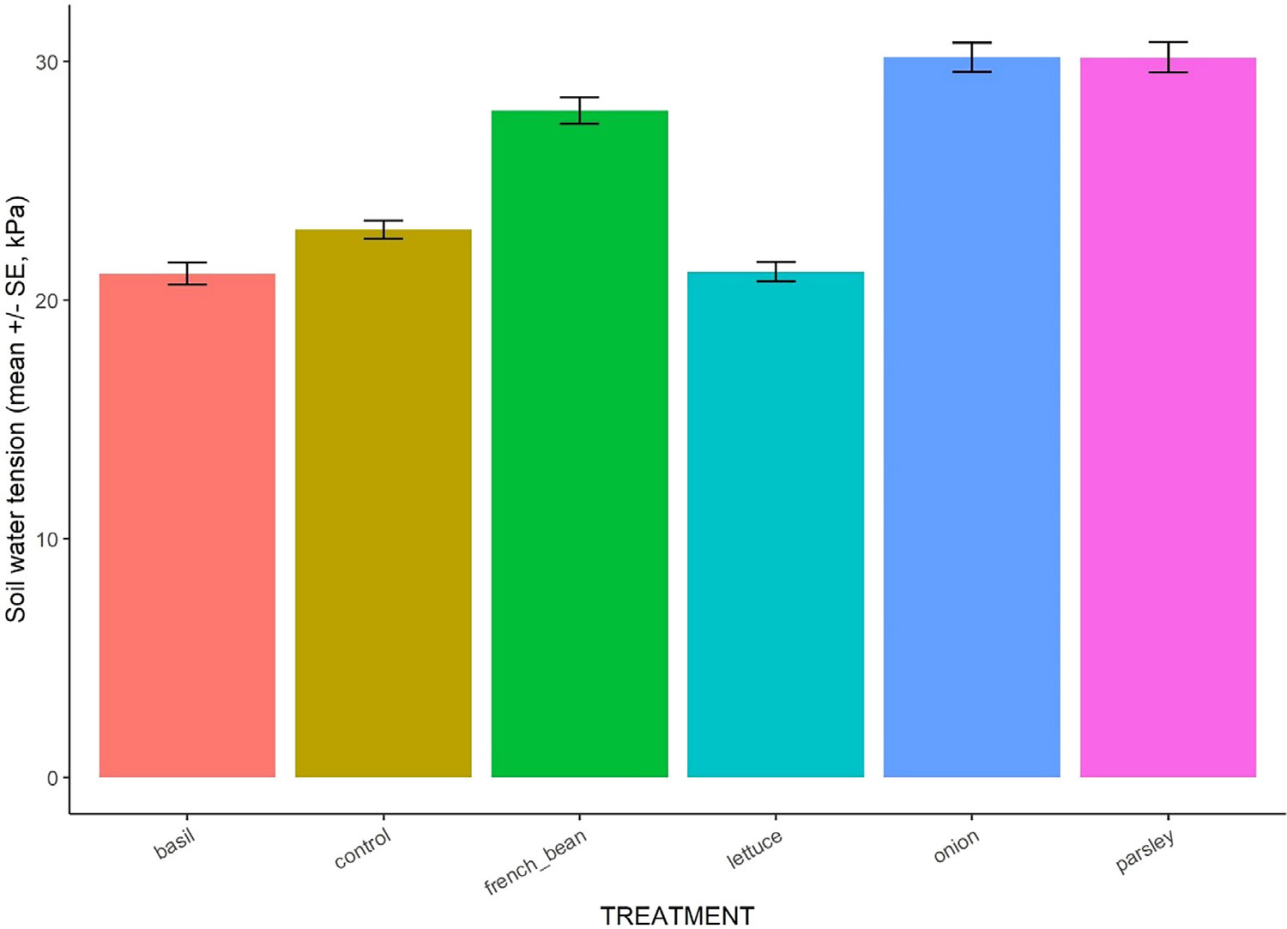
Mean soil water tension in the six treatments. Data were averaged over time (days and years).

**Fig. 3:**
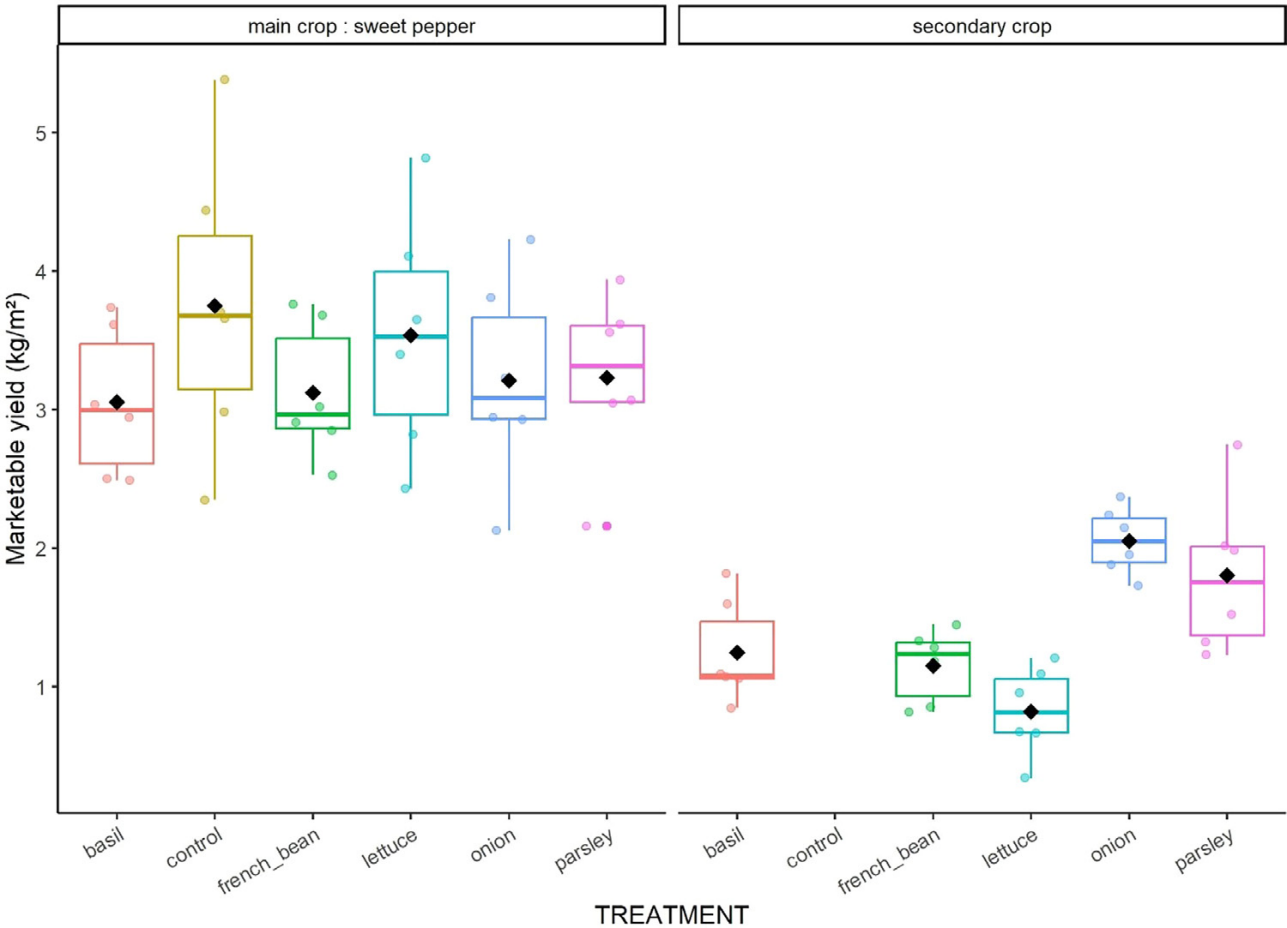
Boxplot of marketable yields of main and secondary crops in the six treatments. Data were pooled over years.

**Fig. 4:**
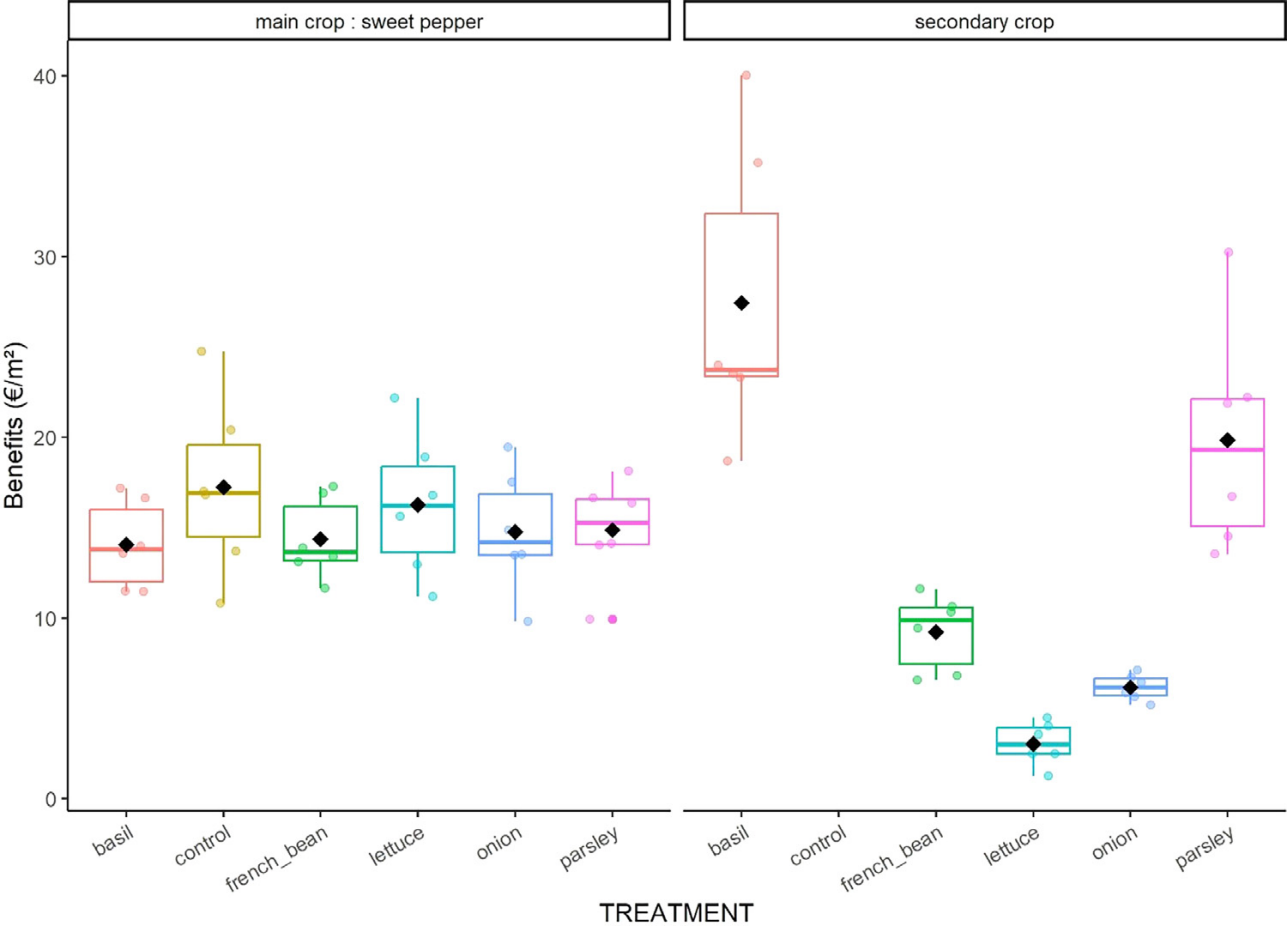
Boxplot of economic benefits of main and secondary crops in the six treatments. Data were mooled over years.

**Fig. 5:**
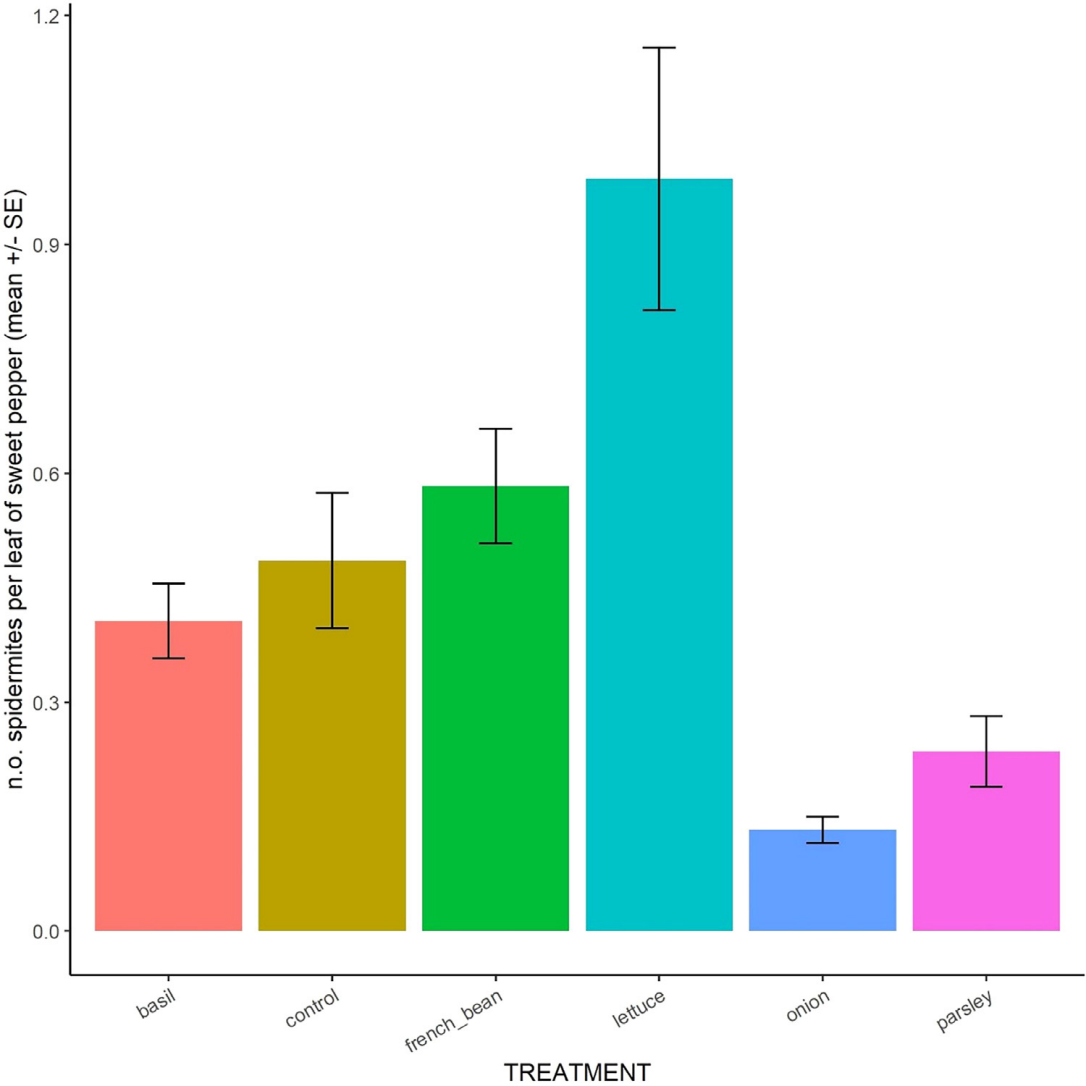
Mean spider mite population on sweet pepper in the six treatments. Data were averaged over time (days and years).

**Fig. 6:**
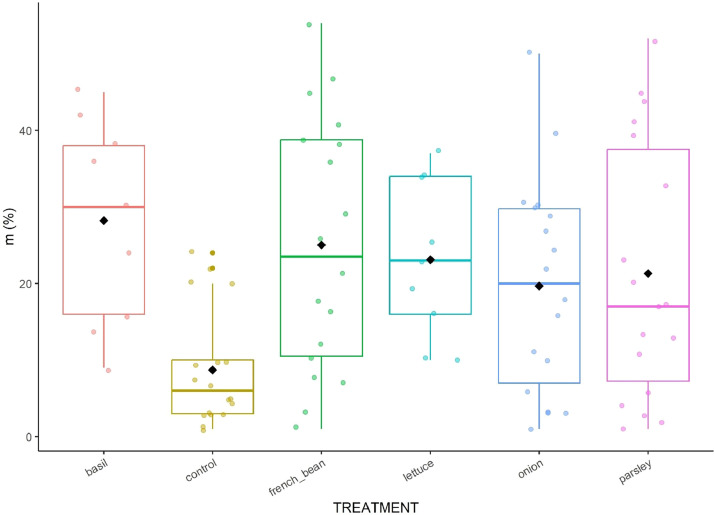
Boxplot of the intensity of the mycorrhizal colonization in the mycorrhized root fragments of sweet pepper in the six treatments. Data were pooled over time (days and years).

**Fig. 7:**
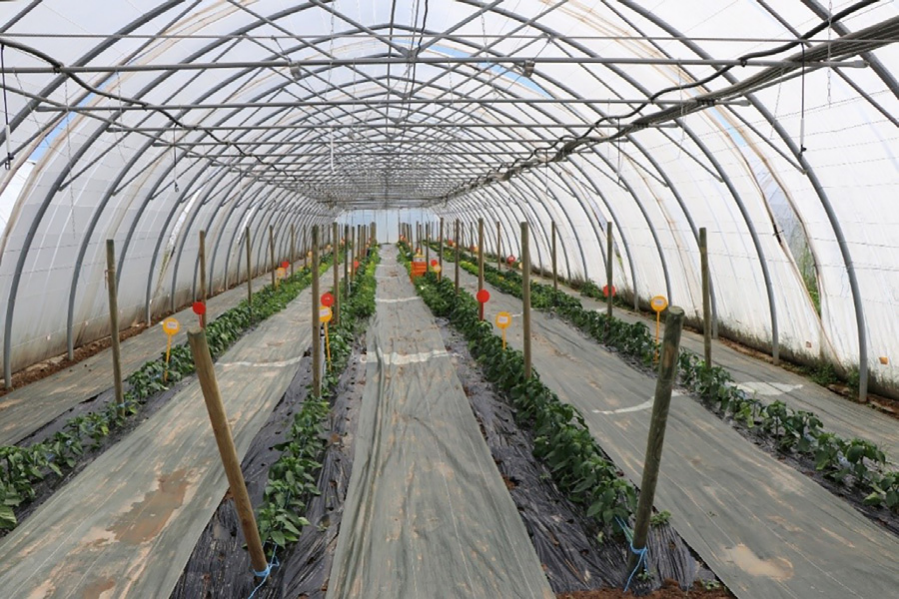
Tunnel view.

**Fig. 8:**
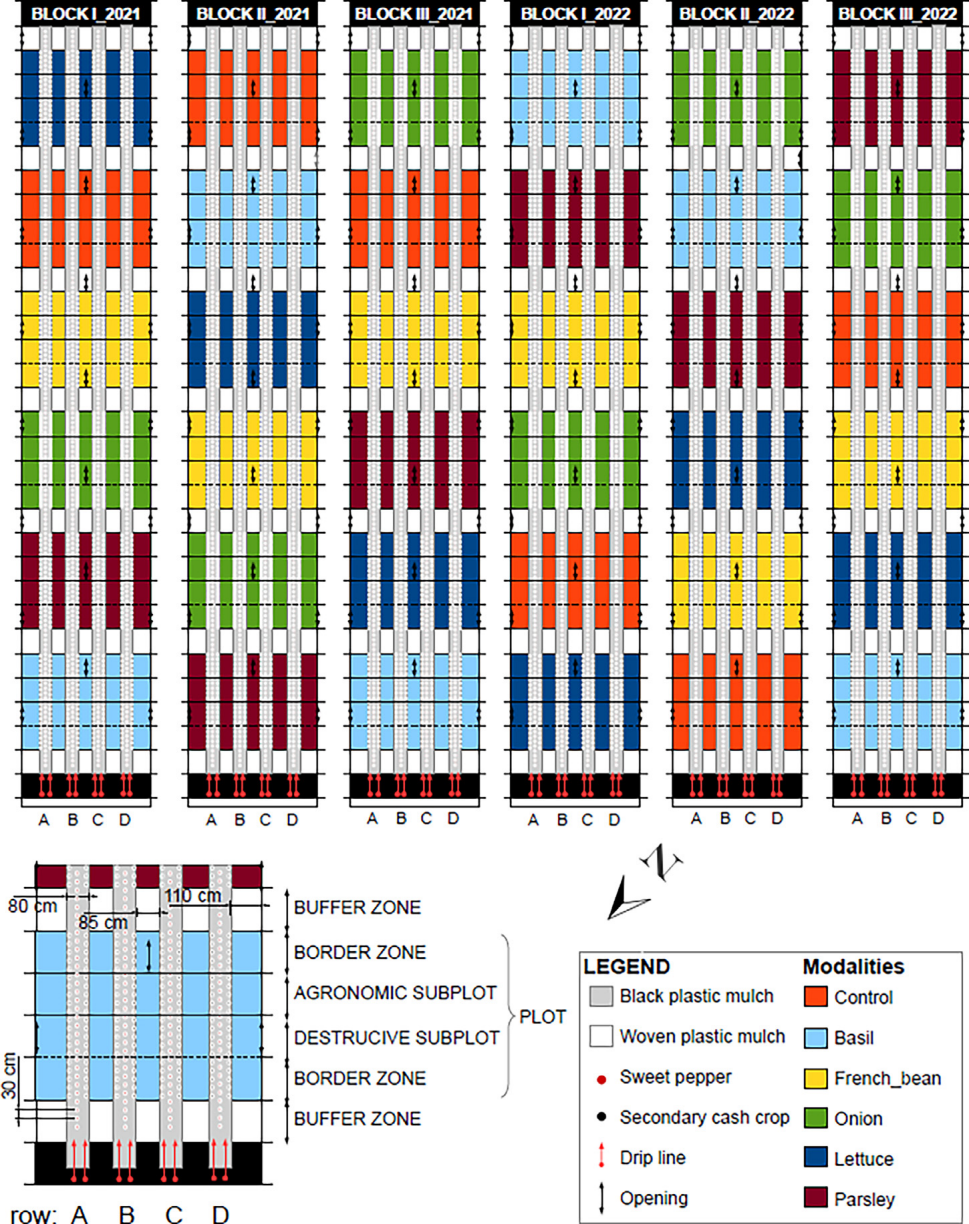
Experimental design, plot description and spatial arrangement of the crops.

**Fig. 9:**
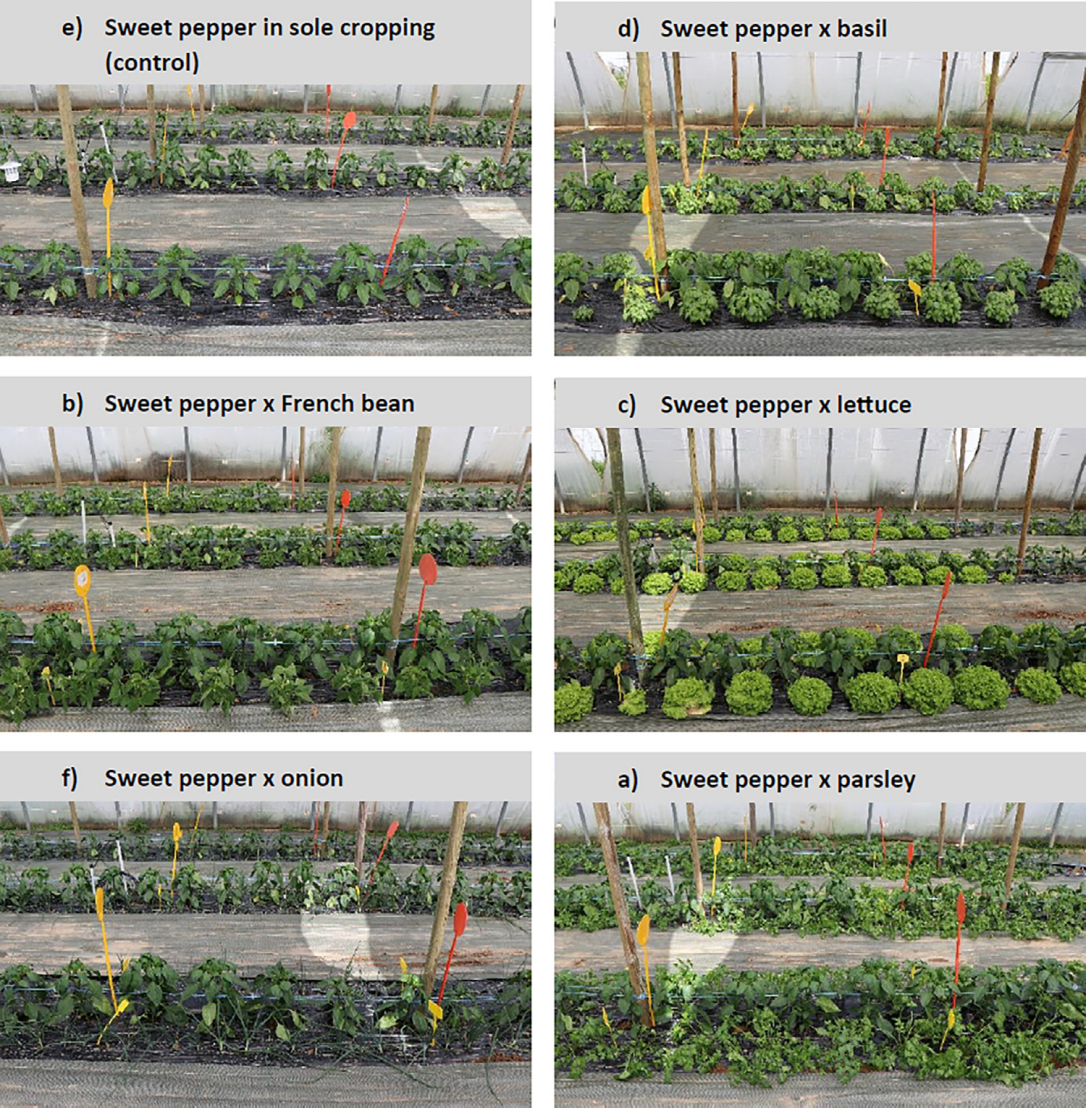
Plant arrangement in the six treatments.

**Fig. 10:**
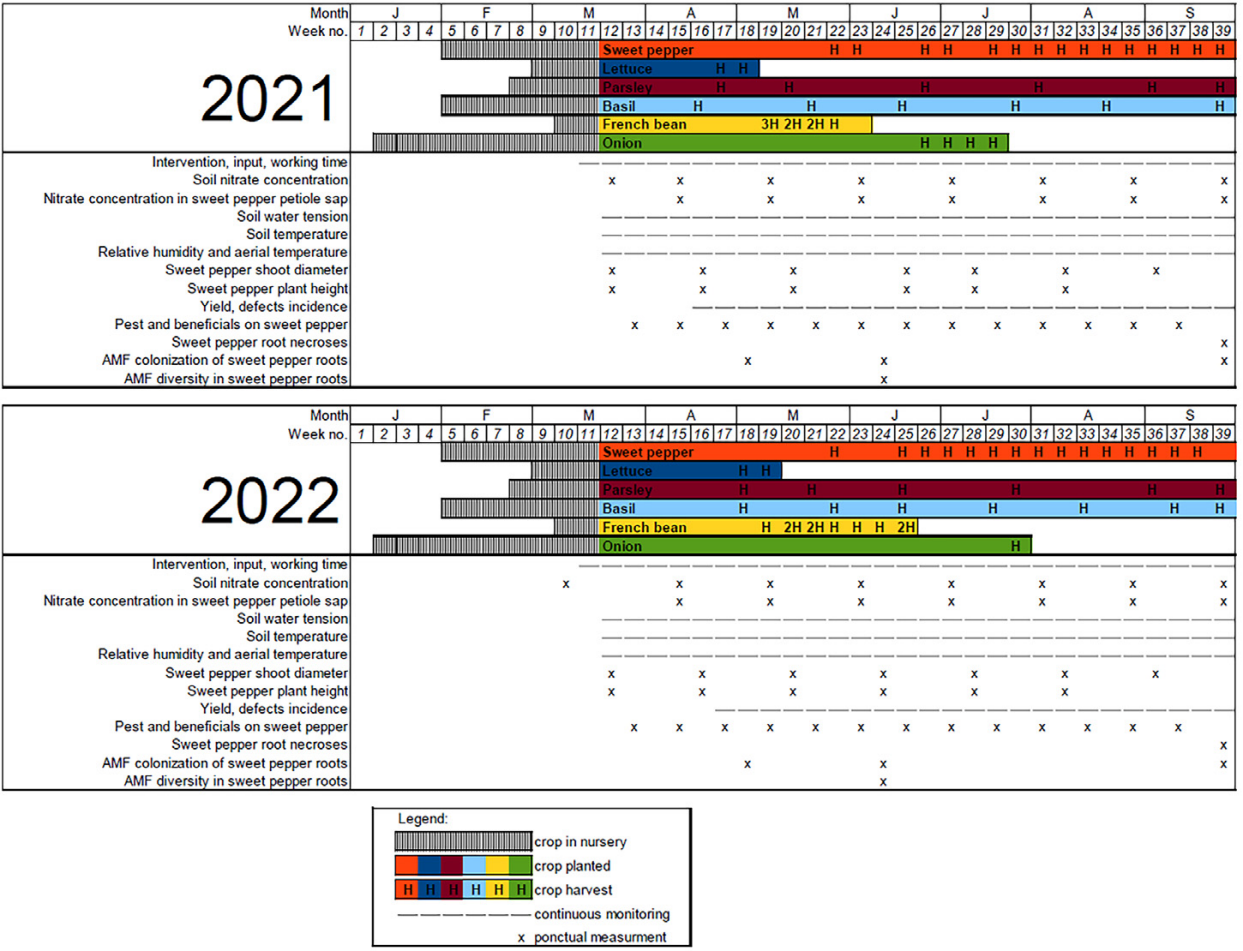
Crop and measurement calendar.

**01_Variables_description.csv:** Explanation for each variable/column used within the dataset.

**02_Cultural_practices_and_economic_costs.csv:** Cultural practices carried out in all treatments and blocks with details on date, inputs (names, amount, unit price), working times, economic costs of inputs and labor. Each line corresponds to a cultural practice carried out for at least one treatment.

**03_Nitrate_concentration_in_soil_and_petiole_sap_of_sweet_pepper.csv:** Raw data of nitrate concentration dynamics in soil (Fig. 1) and of sweet pepper petiole sap in all blocks and treatments.

**04_Irrigation_soil_water_tension_and_climatic_condition.csv:** Synthetized daily data on water amounts provided by trickle and sprinkler in each block, potential evapotranspiration and average of soil water tension for each watermark sensor allocated in the different treatments and blocks (Fig. 2). Data also include minimal, maximal and average values of relative humidity as well as soil and air temperature during daytime and nighttime and over 24 h in each block. Each line corresponds to one date.

**05_Sweet_pepper_growth.csv:** Raw data on the dynamics of sweet pepper stem diameter and plant height in all blocks and for all treatments. Each plant measured was assigned a unique identifier and the row in which it was planted was specified.

**06_Dynamics_of_yield_economic_benefit_and_defect_incidence.csv:** Raw data on the dynamics of marketable, unmarketable and residue yields for each crop species, year, block and treatment. Each line corresponds to the harvest of one crop in one block and for one treatment at one date. This file also includes unit sales prices of products, the economic benefits and the rates of products with defects for 32 different kinds of defects. A defect was noted when it was visible but did not necessarily lead to downgrading the product as unmarketable.

**07_Synthetized_yield_economic_benefit_and_defect_incidence.csv:** Synthetized data on cumulated marketable (Fig. 3), unmarketable and residue yields for each crop cycle, block and treatment. This file also includes unit sales prices, economic benefits (Fig. 4) and the total rates of products with defects for 32 different kinds of defects.

**08_Sweet_pepper_pests_and_beneficials.csv:** Raw data on dynamics of pests and beneficials on sweet pepper across the two cropping cycles. Each line corresponds to one leaf observed at one date. For each leaf, we specified its position on the plant (top or bottom), the plant identifier, the row, the block and the treatment and noted the abundance of six major pests (aphids, spider mites (Fig.5), thrips, whiteflies, leaf miners, leafhoppers) and seven taxa of beneficials (*Aphidoletes aphidimyza*, ladybirds, lacewings, hover flies, *Phytoseiulus persimilis*, other Phytoseiidae and *Feltiella acarisuga*), parasitism rates of aphids and whitefly larvae and the presence of powdery mildew.

**09_Sweet_pepper_root_necrosis.csv:** Raw data on the health status of sweet pepper root systems at crop uprooting. Each line corresponds to one root system observed at the end of one cropping cycle. For each root system, we specified the plant identifier, the row, the block and the treatment and noted a root necrosis index from 0 to 10 as well as the presence of symptoms of *Pyrenochaeta lycopersici* and *Agrobacterium tumefaciens*.

**10_AMF_colonization_rates_of_sweet_pepper_roots.csv:** Raw data on the monitoring of the arbuscular mycorrhizal fungi (AMF) colonization rates of sweet pepper roots (Fig.6). Each line corresponds to one treatment in one block at one date.

**11_AMF_diversity_in_sweet_pepper_roots.csv:** Raw data on the genetic diversity of AMF in sweet pepper roots. Each line corresponds to one amplicon sequence variant (ASV) named in the first column; the 10 following columns describe the number of sequences obtained for each ASV in each root sample. The file also contains the class, order, family, genus, species and virtual taxa assigned to the ASV according to the Maarj*AM* database [2] and the percentage of similarity with the reference sequence of each taxonomic group. The last column contains the reference sequence of each ASV.

To ensure interoperability between all files of the dataset, we used the same column names and data formats for all variables, whether temporal (DATE, YEAR, WEEK, DAP = Days After Planting) or spatial (BLOCK, TREATMENT, ROW, PLANT_ID), for all files.

## 4 Experimental Design, Materials and Methods

### 4.1 Experimental site and design

We carried out the same experiment over two successive years in 2021 and 2022 in southern France in a deep sandy clay loam soil with around 2.2 % organic matter content and a pH of 7.7 under organic farming conditions. For more details on the physicochemical properties of soils, see Table 1.

**Table 1:** Physicochemical soil properties.

Parameter	Block I	Block II	Block III	Unit	Standards
Bulk density	1.3	1.3	1.3	t/m^3^	NF ISO 11465
Available water capacity (AWC)	59	59	59	mm	Calculation
Clay	115	114	108	‰	NF X31-107
Fine silt	252	236	224	‰	NF X31-107
Coarse silt	251	218	231	‰	NF X31-107
Fine sand	266	279	273	‰	NF X31-107
Coarse sand	115	152	164	‰	NF X31-107
Organic matter (OM)	2	2.4	2	%	NF ISO 14235
C/N	9.4	12.1	10.9	unitless	Calculation
Mineralizable N	72	69	66	kg.ha^−1^	Estimation
pH (water)	7.8	7.7	7.6	unitless	Internal method
Total limestone	2	2	<1	g.kg^−1^	NF ISO 10693
CaO	3.37	3.02	3.05	g.kg^−1^	NF ISO 10693
CEC (Metson)	8.9	8.1	8.3	cmol+.kg^−1^	NF X 31-130
P_2_O_5_ (Olsen)	40	40	60	mg.kg^−1^	NF ISO 11263
K_2_O	130	150	130	mg.kg^−1^	NF X 31-108
MgO	400	350	370	mg.kg^−1^	NF X 31-108
Copper EDTA	55	58	62	mg.kg^−1^	NF X31-120
Manganese EDTA	69	65	53	mg.kg^−1^	NF X31-120
Iron EDTA	54	54	67	mg.kg^−1^	NF X31-120
Zinc EDTA	4	3	4	mg.kg^−1^	NF X31-120
Sodium	120	110	120	mg.kg^−1^	NF X 31-108

For each block, 45 soil samples were collected at 0–30 cm depth with an auger on 19/10/2020 and mixed together before physicochemical analysis. Analyses were performed by Aurea laboratory according to their usual procedure.

The experimental design consisted of a randomized complete block with three blocks, each one in an unheated walk-in plastic tunnel (8 × 50 m, Figs. 7, 8), and six treatments: sweet pepper intercropped with basil, French bean, lettuce, onion, parsley and sweet pepper in sole cropping (control). Each replication occupied a tunnel section of 48 m² (8 × 6 m) called a “plot” and was separated from other treatments by a buffer section of 12 m² (8 ✗ 1.5 m) cropped with sweet pepper in sole cropping. Treatments were randomly assigned to plots each year. In each plot, we collected data on the central area composed of an “agronomic” and a “destructive” subplot, each one measuring 13.3 m² (8 × 1.66 m) (Fig. 8). “Destructive subplots” were used for all variables for which acquisition methods were susceptible to disturb soil and plants, namely nitrate concentration in soil and in petiole sap of sweet pepper, soil water tension, AMF colonization rates and AMF diversity in sweet pepper roots. All other variables were acquired in “agronomic subplots.” Fields were previously cultivated with multi-cut sorghum green manure for 7 months in summer and autumn 2020 for homogenization followed by an occultation with a silage tarp for 4 months in winter 2020. Fields were cultivated with rye–vetch mixture as green manure followed by lettuce in sole cropping between the two experiments to meet organic farming specifications.

### 4.2 Plant material, cropping calendar, arrangement and conditions

Sweet pepper (“Achille”, HM.Clause), French bean (“Oreo”, Vilmorin), onion (“Rouge de Toulouges”, Agrosemens) and parsley (“Gigante d'Italia,” Voltz) were grown in nursery before being transplanted in tunnels. The intercropping design was additive with the same sweet pepper density (D = 1.5 plants.m^−2^) for all treatments; sweet pepper plants were arranged in four simple rows (named A, B, C and D) 1.65 m apart with a plant spacing of 0.33 m within the row. For intercropping treatments, two rows of the secondary crop were planted at 0.33 m from both sides of each row of sweet pepper with a plant spacing of 0.11 m for onion (D = 9 plants.m^−2^) and 0.33 m for basil, lettuce, French bean and parsley (D = 3 plants.m^−2^) (Figs. 8, 9). For details on the crop calendar, see Fig. 10.

Irrigation, crop protection and other regular cultural practices were applied uniformly throughout all experimental plots as needed during cultivation. Soils were supplemented with organic fertilizers as base dressing taking into account the soil chemical analysis to reach the following rates: 200 N − 50 P − 300 K + 30 MgO at the beginning of the experiments. Each planting bed, composed of one row of sweet pepper and two rows of a secondary crop, was covered by a black plastic mulch and irrigated with two trickle lines located between rows of the main and secondary crops. Irrigation was scheduled to compensate for the evapotranspiration loss from sweet pepper in sole cropping (Kc_sweet pepper_ × EPT). Alleys between planting beds were covered by a woven plastic mulch to ensure weed control. Sweet pepper plants were trellised in hedges with wooden posts and horizontal strings; all side shoots under the first flower were removed.

### 4.3 Measurements, observation and sampling

The crop and measurement calendar were made as consistent as possible between the two years of the experiment, but a few time shifts did occur (Fig. 10).

We registered the cultural practices carried out in each block and treatment throughout the cropping cycles. For each intervention, we specified the date, the crops concerned, the names, amounts of inputs with their corresponding units, the pest targeted when appropriate and the working time (in h.m^−2^). We also calculated input and labor costs (in €.m^−2^) and specified unit prices of inputs with their corresponding units and hourly labor costs (in €/h^−1^) used to calculate them.

We monitored nitrate concentration in soil (S_NC) and in sweet pepper petiole sap (SP_NC) every 4 weeks (Fig. 10**)**. For each sampling date, we collected 8 soil samples at 0–30 cm depth with an auger and petioles of 16 young and fully expanded leaves in each “destructive subplot” on 4 sweet pepper rows. We mixed soil samples, sieved them at 4 mm and analyzed the soil suspension (100 g of fresh soil in 100 ml of water) with a nitrate strip (RQflex® 10, Merck) and corrected the soil nitrate concentration by the soil water content estimated through the soil core moisture. We chopped the petioles and pressed them with a hydraulic press to extract plant sap, which we analyzed immediately with a nitrate strip (RQflex® 10, Merck). S_NC and SP_NC were expressed in kg.ha^−1^ and mg.L^−1^, respectively.

We measured daily amounts of water provided by trickle (TIA) and sprinkler (SIA) in each block with water meters and expressed them in L.m^−2^.day^−1^.

We monitored the soil temperature (ST) through the cropping cycles in each block with a PT1000-type temperature sensor placed at 20 cm depth in the middle of the sweet pepper rows “B”. Air temperature (AT) and relative humidity (RH) were monitored in each block with thermo-hygrometers (EE08, E+E Elektronik®) placed in the sweet pepper canopy. All records were collected by a data acquisition module (ADAM, 4000 series) programmed to collect data every 20 s. Then we extracted 9 derived variables from each parameter registered: their minimal, maximal and average values during daytime and nighttime and over 24 h. We expressed ST and AT in°C and RH in %. The daily evapotranspiration expressed in L.m^−2^ was calculated according to the De Villele formula described in Hammami et al. [3] based on climatic data collected by a weather station located in the vicinity of the tunnels (42.638; 2.970; alt 10 m). This formula is adapted for conditions under plastic tunnels.

We monitored the soil water tension (SWT) through the cropping cycles with two to three tensiometer sensors (Watermarks®) placed in each “destructive subplot” at 20 cm depth on sweet pepper rows “B” and programmed to collect data every 4 h. These data were then averaged by day for each tensiometer sensor and expressed in kPa.

We measured sweet pepper stem diameters at the collar (SP_SD) with an electronic caliper (IP67, TESA) and plant height (SP_PH) with a measuring tape every four weeks, on the 20 plants of each “agronomic subplot”. SP_SD and SP_PH were expressed in mm and cm, respectively.

We estimated yields of sweet pepper and secondary crops by collecting products from “agronomic subplots” through the crop cycles. Products were then classified as marketable or unmarketable according to organic short supply chain standards, counted and weighed; we also collected and weighed the aerial part of secondary crop residues at the end of their crop cycle. The number and weight of products were then added up for each cropping cycle and divided by the agronomic subplot area to obtain yields expressed in no.m^−2^ and kg.m^−2^ for the different categories: marketable (NMP, MY), unmarketable (NUP, UY) and residue (RY). We calculated the economic benefits (BENEFITS in €.m^−2^) of each crop by multiplying MY by the unit sales price of the product observed in the local organic farming short supply chain (UNIT_PRICE in €.kg^−1^). We also listed all visual defects of each product and added them together for each cropping cycle to calculate an incidence rate for each defect and each crop.

We monitored pests and beneficials on sweet pepper every two weeks by observing 2 leaves per plant on the upper and lower part of 20 plants from each “agronomic subplot”. For each leaf we noted the abundance of 6 pest taxa (aphids, spider mites, thrips, whiteflies, leaf miners, leafhoppers) and 7 beneficial taxa (*Aphidoletes aphidimyza*, ladybirds, lacewings, hover flies, P*hytoseiulus persimilis*, other Phytoseiidae and *Feltiella acarisuga*), parasitism rates of aphids and whitefly larvae and the presence of powdery mildew.

We observed sweet pepper root necrosis at crop uprooting on 20 plants from each “agronomic subplot”. We extracted root systems from the soil with a beaker fork, rinsed them with water and scored by visual observation the proportion of the root surface affected by necrosis on a 0–10 scale (RNI, 0: 0 %; 1: 1–10 %; 2: 11–20 %; etc. 10: 91–100 % root surface affected by necrosis) adapted from Zeck [4]. We also noted the presence of visually identifiable symptoms induced by *Pyrenochaeta lycopersici* and *Agrobacterium tumefaciens* on root systems.

AMF colonization rates in sweet pepper roots were determined at 4 dates per year on 4 treatments in 2021 (control, French_bean, onion and parsley) and on all treatments in 2022. We collected a composite sample of 18 root fragments of 1 cm length on 12 plants in each “destructive subplot”. Root fragments were then colored according to Vierheilig et al. [5], rinsed with lactoglycerol and observed with a microscope (×200–800). The following mycorrhizal colonization rates were estimated according to Trouvelot et al. [6] using Mycocalc software (INRAE, Dijon) by Mycea SAS: mycorrhization frequency in roots (F), intensity of the mycorrhizal colonization in the total root system (M) and in the mycorrhized root fragments (m), arbuscule abundance in the total root system (A) and in mycorrhized root fragments (a), vesicular abundance in the root system (V) and in mycorrhized root fragments (v). Mycorrhizal colonization rates were expressed in %.

Mycea SAS determined the genetic diversity of AMF in roots of sweet pepper at 1 date per year on 14/06/2021 and 14/06/2022. This analysis concerned only the onion, parsley, French bean and control treatments in 2021 and all treatments in 2022. For each date and treatment, we collected a composite sample of 5 g of young sweet pepper roots on 12 plants in “destructive subplots” of the three blocks (one sample per treatment). DNA was extracted from collected roots with a “Fast DNA kit for soil” amplified using 18S AMF specific primers and sequenced with the next-generation sequencing (NGS) method. Species represented by fewer than 100 sequences were not considered in order to focus on the most significantly present species. Sequences were grouped by similarity (∼97 % similarity). The taxonomic assignment was carried out according to the Maarj*AM* database [2].

## Ethics Statements

The study did not involve work with humans, animals, or sensitive information.

## CRediT authorship contribution statement

**B. Perrin:** Conceptualization, Methodology, Validation, Formal analysis, Data curation, Writing – original draft, Writing – review & editing, Visualization, Supervision. **C. Leroy:** Investigation, Validation. **L. Parès:** Methodology, Investigation. **P. Pradere:** Investigation. **M. Goude:** Investigation. **B. Salvador:** Investigation. **T. Marrec:** Resources. **L. Comes:** Investigation, Validation. **R. Huot-Marchand:** Investigation, Validation. **E. Guillot:** Writing – review & editing. **A. Lefèvre:** Writing – review & editing, Funding acquisition.

## Data Availability

Experimental dataset of the impact assessment of vegetable intercropping on agroeconomic performances, pests and beneficials, and soil resources (Original data) (recherche.data.gouv.fr)
